# Assessing the benefits of using mate-pairs to resolve repeats in de novo short-read prokaryotic assemblies

**DOI:** 10.1186/1471-2105-12-95

**Published:** 2011-04-13

**Authors:** Joshua Wetzel, Carl Kingsford, Mihai Pop

**Affiliations:** 1 Department of Computer Science, Princeton University, Princeton, NJ, USA; 2 Center for Bioinformatics and Computational Biology, University of Maryland, College Park, MD, USA

## Abstract

**Background:**

Next-generation sequencing technologies allow genomes to be sequenced more quickly and less expensively than ever before. However, as sequencing technology has improved, the difficulty of *de novo *genome assembly has increased, due in large part to the shorter reads generated by the new technologies. The use of mated sequences (referred to as mate-pairs) is a standard means of disambiguating assemblies to obtain a more complete picture of the genome without resorting to manual finishing. Here, we examine the effectiveness of mate-pair information in resolving repeated sequences in the DNA (a paramount issue to overcome). While it has been empirically accepted that mate-pairs improve assemblies, and a variety of assemblers use mate-pairs in the context of repeat resolution, the effectiveness of mate-pairs in this context has not been systematically evaluated in previous literature.

**Results:**

We show that, in high-coverage prokaryotic assemblies, libraries of short mate-pairs (about 4-6 times the read-length) more effectively disambiguate repeat regions than the libraries that are commonly constructed in current genome projects. We also demonstrate that the best assemblies can be obtained by 'tuning' mate-pair libraries to accommodate the specific repeat structure of the genome being assembled - information that can be obtained through an initial assembly using unpaired reads. These results are shown across 360 simulations on 'ideal' prokaryotic data as well as assembly of 8 bacterial genomes using SOAPdenovo. The simulation results provide an upper-bound on the potential value of mate-pairs for resolving repeated sequences in real prokaryotic data sets. The assembly results show that our method of tuning mate-pairs exploits fundamental properties of these genomes, leading to better assemblies even when using an off -the-shelf assembler in the presence of base-call errors.

**Conclusions:**

Our results demonstrate that dramatic improvements in prokaryotic genome assembly quality can be achieved by tuning mate-pair sizes to the actual repeat structure of a genome, suggesting the possible need to change the way sequencing projects are designed. We propose that a two-tiered approach - first generate an assembly of the genome with unpaired reads in order to evaluate the repeat structure of the genome; then generate the mate-pair libraries that provide most information towards the resolution of repeats in the genome being assembled - is not only possible, but likely also more cost-effective as it will significantly reduce downstream manual finishing costs. In future work we intend to address the question of whether this result can be extended to larger eukaryotic genomes, where repeat structure can be quite different.

## Background

Next-generation sequencing platforms such as Illumina, ABI Solid, and 454 allow genomes to be sequenced more quickly and at a lower cost than ever before. However, as sequencing technology has improved, the wealth of available data has not made genome assembly easier. In fact, the level of difficulty has increased, as the cost savings provided by new technologies are accompanied by a reduction in achievable read-length. Assembly software is now faced with the task of assembling reads ranging from approximately 35 to 500 nucleotides, in contrast to the 800-2000 nucleotide reads previously generated by the traditional Sanger technology. Additionally, the error characteristics of the newer technologies are not as well known as they were for traditional Sanger sequencing. *De novo *assemblies (even in the case of prokaryotic genomes) are highly fragmented [1]. In addition, advances in sequencing technologies have not been mirrored by corresponding improvements in finishing - a time-, labor-, and cost-intensive process aimed at reconstructing a complete, gapless, genome sequence from a fragmented assembly. As a result, the majority of genomes remain in an incomplete 'draft' state, hampering studies that rely on long-range genome structure information (e.g., analysis of operon/regulon structure, or large-scale genomic variation).

Genomic repeats - DNA segments repeated in nearly-identical form throughout a genome - are the main reason why genome assemblers cannot automatically reconstruct complete genome sequences from modern sequencing data. Repeats have a number of biological causes, including transposable sequence elements (which can often be highly abundant in a genome), prophages, highly-conserved gene clusters (e.g., the Ribosomal RNA operons in bacteria), or large segmental duplications, and can vary from short simple sequence repeats (e.g., AAAAA, ACACAC) to long stretches of DNA (thousands to tens of thousands of base-pairs) that are highly or completely identical. In the context of inexpensive next-generation sequencing and assembly, some of the original pitfalls of genome assembly, such as coverage gaps and confusion caused by read errors, can often be obviated by simply sequencing the genome to very high coverage levels. Repeated sequences, on the other hand, cannot be avoided by simply over-sequencing, and lead to considerable ambiguity in the reconstruction of a genome, providing limits on the length of the contiguous DNA segments (contigs) that can be correctly reconstructed using unpaired reads [2].

Graph-theoretic models of genome assembly provide an effective framework for analyzing the impact of repeats on the complexity of genome assembly [3]. The genome assembly problem is commonly formulated as finding a constrained path through an appropriately-defined graph. Throughout this article we will rely on an Eulerian formulation [4] that reduces the assembly problem to finding a Chinese Postman path/tour (a minimum length path through the graph that covers all edges) within a de Bruijn graph (to be defined later). Under this formulation, repeats appear as forks in the graph that make it difficult to select the graph traversal that corresponds to the correct reconstruction of the genome from among an exponential (in the number of repeats) number of possible Chinese Post-man paths. Without additional information, genome assemblers can only correctly reconstruct relatively short, unordered segments of the genome (corresponding to those sections of the graph which contain no forks).

In addition to the collection of reads, most sequencing technologies also produce pairwise constraints on the placement (approximate distance and relative orientation) of these reads along the genome - mate-pair information. This information can further constrain the possible traversals of the assembly graph, thereby allowing longer segments of the genome to be unambiguously reconstructed. Mate-pair information has been a critical component of most genome projects, starting with *Haemophilus influenzae *[5] - the first free-living organism to be fully sequenced. Most genome assemblers now include modules that can use mate-pair information for scaffolding and repeat resolution (Celera Assembler [6], Velvet [7], Euler [3], Arachne [8], and ALLPATHS [9] to name just a few), and stand-alone scaffolding tools such as Bambus [10] allow the incorporation of mate-pair data into virtually any assembly [11]. Furthermore, mate-pairs are frequently used for the purpose of assembly validation in tools such as BACCardi [12], Consed [13], and Hawkeye [14].

Most of the research on the use of mate-pair information in genome projects has focused primarily on the use of this information to achieve long-range connectivity by spanning long repeats and gaps in the assembly due to insufficient sequencing coverage. As a result, substantial efforts have been focused on the development of robust protocols for constructing long-range (8-10 kbp or longer) mate-pair libraries. Here, we explore a complementary purpose for mate-pairs - the automatic resolution of repeats during assembly. We argue that, due to affordable high-throughput sequencing technologies, coverage gaps are far less frequent than they used to be, and assembly fragmentation is primarily caused by repeats. Thus improvements in repeat resolution algorithms will translate into substantial improvements in the quality of the resulting assemblies.

### Resolving repeats with mate-pairs

All previously published techniques for repeat resolution rely on the same basic observation: If a unique path in the assembly graph can be found that connects the sequences at the ends of a mate-pair and the length of this path matches the approximately known mate-pair size, then this path can be inferred to be correct; i.e., the path represents a partial traversal of the graph that is consistent with the correct reconstruction of the genome being assembled. Since the problem of finding a path of predefined length through a graph is *NP*-hard [15], the algorithms used during assembly rely on various heuristics for efficiently finding paths that support mate-pair information.

The EULER assembler [3] greedily finds paths whose lengths are consistent with the size of mate-pairs (the authors indicate that such paths can be easily found for a majority of mate-pairs), then converts these paths into artificial long reads that can be processed through the Eulerian superpath algorithm [4]. Conflicts between paths through the graph are resolved by prioritizing paths on the basis of their support (number of mate-pairs that confirm a given path) [16]. The Velvet [7,17] and ALLPATHS [9] assemblers take into account the uniqueness of nodes in the assembly graph. Specifically, mate-pair links are considered only if they are anchored in contigs that correspond to non-repeated sequences in the genome (usually determined through depth of coverage statistics).

Analyses of the effectiveness of such algorithms have typically assumed the parameters of the sequencing experiment to be fixed; i.e., the goal is to build the best assembly possible given the types of data commonly generated in current sequencing projects. An exception is the study by Chaisson *et al*. [16] where the authors evaluate the effect of read length and read quality on the ability to reconstruct a genome. In other words, they assume one can tune the read length generated by a sequencing machine (which is a realistic assumption for the Illumina technology) and estimate whether the assembly improves, and by how much, if the length of reads is increased. Such analyses are critical for providing a scientific basis for picking the optimal trade-off between sequencing cost (which increases with the read length) and quality of assembly.

In our work, we pose a complementary question: How useful are mate-pairs for resolving repeats in *de novo *assemblies created from short-reads? Which types of mate-pair libraries most effectively resolve repeats and minimize the amount of manual finishing needed to complete the genome (given that there is some restriction on the number of mate-pair libraries that can be cost-effectively produced in a real sequencing experiment)? Similar questions have been addressed by recent publications in the context of sequence alignment. Chikhi *et al*. [18] evaluate how read length affects genome resequencing experiments, and Bashir *et al*. [19] developed a method to identify the mate-pair sizes that are optimal for detecting structural variation through mapping. However, the question of how effective mate-pairs are for the resolution of repeats and how this parameter of the sequencing experiment can be adjusted to improve assemblies has not previously been analyzed in a systematic fashion.

### Measuring assembler performance

Metrics commonly used for comparing the quality of genome assemblies are primarily focused on statistics derived from the global distribution of contig sizes. Statistics such as the number of contigs, average contig size, and N50 contig size are frequently reported in the literature. (N50 contig size is the size *c *for which 50% of the bases in the genome are contained in contigs of size at least *c*.)

When the correct answer is known (e.g., reassembly of a known genome), one can also record the number of errors found in the assembly. An alternative approach is proposed by Chaisson *et al*. [16] where they compare the results of an assembly to a theoretical optimal: the best assembly that can be reconstructed without errors from a genome, given the repeat graph (see Methods) of that genome.

In our study we are specifically targeting this theoretical optimum: in an idealized setting (perfect sequencing data), what is the best possible assembly that can be obtained given the parameters of the sequencing process? While aiming for this theoretical optimum, we also maintain a dose of reality about certain aspects of sequencing projects, restricting ourselves to the use of only two mate-pair libraries (a fairly standard procedure due to costs) and use of read-lengths that are reflective of inexpensive next-generation sequencing. Like Chaisson *et al*. [16] and Kingsford *et al*. [2], we start with the idealized repeat graph of a genome (see Methods), the structure of which is determined by the read length. Although we are attempting to maximize the size of contigs that can be unambiguously reconstructed from this graph, contig size statistics are difficult to compare across genomes and may not adequately describe the amount of repeat resolution that a set of mate-pairs provide. Therefore, we focus instead on a measure of assembly ambiguity that is directly related to unresolved repeats. Specifically, we measure the number of manual experiments that would be necessary to completely resolve the structure of a genome during finishing. Briefly, a path through the repeat graph can be uniquely determined by pairing up the edges adjacent to repeat nodes. This pairing is commonly determined during finishing through targeted PCR experiments.

We measure the usefulness of a mate-pair library in terms of the amount of manual finishing effort that can be saved through its use (see Methods for details). We show that, in the context of high-depth prokaryotic sequencing experiments, very short mate-pairs are more useful than long mate-pairs (sizes as high as 40,000 bp are often generated in sequencing projects) for resolving repeats, and that choosing mate-pair sizes based on the repeat structure (defined in Methods) of the assembly graph is a powerful approach for creating more complete assemblies. Our results hold across 360 'ideal' sequencing experiments (see following section) as well as when using an off-the-shelf assembler in the presence of base-call errors (assembly of 8 bacterial genomes using SOAPdenovo [20]). These results can serve as a basis for developing new algorithms and sequencing strategies that will improve *de novo *assemblies. Due to computational efficiency considerations we limited our analysis to prokaryotic genomes. Whether or not our results can be extended to eukaryotes (whose repeat structure is often quite different from prokaryotes) remains an exercise for future work.

## Results

We simulated ideal sequencing projects (no gaps, no errors, known mate-pair sizes) for 391 complete bacterial genomes using a range of read lengths and mate-pair sizes. Read lengths were chosen to be reflective of reads that can be affordably obtained using next-generation sequencing platforms. In addition, for 360 of these genomes, we performed a direct comparison of the amount of repeat resolution that can be obtained when applying information provided by long mate-pairs (libraries between several thousands to tens of thousands of basepairs are commonly generated in current sequencing experiments) vs. mate-pairs whose sizes are 'tuned' to the repeat structure of the genomes being assembled. Finally, we show that the results of our simulated comparison are recapitulated when assembling 8 bacterial genomes in the presence of base-call errors using an off -the-shelf assembler (SOAPdenovo).

We assume a refined version of the repeat graph for a genome; specifically, the graph that can be obtained through a series of lossless transformations on the original de Bruijn graph (see Methods). Since all of the transformations used simply convert unambiguous paths from the original graph into single nodes, the resulting simplified structure is equivalent (in terms of the spectrum of possible graph traversals) to the original graph. The simplified version of the graph is more compact and better highlights the complexity introduced by repeats, since every node in the simplified graph is either a repeat itself or is one of a set of possible nodes to traverse in between a pair of repeats. (For more information on this graph refinement process, see Methods and a more in depth description in an article by Kingsford *et al*. [2].)

Throughout most of our analysis, we consider *k*-mer size (the size of sequences used in initial construction of the de Bruijn graph) and read-length to be interchangeable, denoting both with the letter *k*. Although read-length and *k*-mer size are not typically the same in a real sequencing and assembly experiment (in reality longer reads are decomposed into shorter *k*-mers), we consider the distinction to be irrelevant throughout much of the discussion of our simulations; the only exception being the discussion of SOAPdenovo assemblies. Since our 'idealized' graphs (see Methods) are constructed from complete genomes decomposed directly into *k*-mers, we implicitly imagine that we have perfect reads of length *k *and that they can be used in their entirety during graph construction. Since a read of length *k *can provide a *k*-mer of length at most *k*, ignoring the distinction still provides a valid (if perhaps lenient) upper-bound on what one can expect to achieve if using reads of length *k *with a real assembler.

Our results begin with some theoretical analysis of the usefulness and limitations of mate-pairs and then proceed to an empirical investigation into the benefits of 'tuning' mate-pair libraries to target repeats of high complexity. It should be noted here that due to limitations of our current implementation to handle larger, eukaryotic de Bruijn graphs, we have restricted the scope of our results to cover only assembly of prokaryotic genomes. We intend to explore whether or not these results can be extended to eukaryotic assemblies in future work. A complete set of data files produced for this experiment is available at: http://www.cbcb.umd.edu/~wetzeljo/matePairs/.

### Performance of shortest-path heuristic

Our study relies on a simple heuristic for the use of mate-pair information: mate-pairs are only used if an unambiguous shortest path through the assembly graph connects its end-points and is consistent with the insert length. Medvedev *et al*. [21] use a similar shortest-path approach in their maximum likelihood genome assembler. While more elaborate approaches have been proposed (e.g. searching for the 'best' path from among multiple paths that connect the endpoints of a mate-pair [9]), such methods are computationally expensive. Furthermore, we find that the length of a shortest path connecting the two ends of a mate-pair exactly matches the insert length almost 90% of the time, even for relatively long (8 kbp) libraries and short reads (35 bp) (see Table 1). For longer reads and shorter mate-pairs, this statistic approaches 100%. The shortest-path heuristic is less effective only when using very long mate-pair inserts (35 kbp) with short reads (35-50 bp). In this case, as few as 62% of the mate-pairs correspond to a shortest path.

**Table 1:** Mate-pair statistics

Read/MPLen(nt)	Short.Path(%)*^a^*	Usable(%)*^b^*	Compl.Red(%)*^c^*
100/400	99.96	0.48	57.97
100/8000	95.85	2.69	60.13
100/35000	84.60	6.55	58.27

35/400	99.5	1.06	35.87
35/8000	87.84	6.77	33.66
35/35000	62.29	10.78	31.62

A table summarizing average (mean) statistics across 391 prokaryotic genomes (see Additional file 1) when applying 50,000 mate-pairs of a particular length to a graph constructed from *k*-mers of a particular length. *^a^*Percentage of mate-pairs that can be mapped to a shortest path in the graph when comparing exact length of the insert to length of the path. *^b^*Percentage of mate-pairs that can ultimately provide unambiguous information for repeat resolution (see Methods on repeat resolution). *^c^*Percent reduction in finishing effort (see Methods on finishing complexity) that the set of mate-pairs can provide.

While a large fraction of mate-pairs could, in principle, be used during assembly through the shortest-path heuristic, the vast majority of mate-pairs do not span repeat nodes, and many cannot yield unambiguous paths (see Methods). Thus, most mate-pairs are not useful for repeat resolution. For example, on average only 0.5% of all mate-pairs from a 400 bp library could be used to resolve repeats (as outlined in Methods) in assemblies constructed from 100 bp reads (see Table 1). This result is unsurprising when the mate-pair length is short with respect to the read-length, as both ends of the mate-pair are usually found within the same node of the assembly graph. However, even in the case when mate-pairs are significantly longer than the read length, only a small fraction of the mate-pairs are ultimately usable (e.g., 6.77% when using 35 bp reads and mate-pair library of 8 kbp insert size). An intuitive explanation for this phenomenon is the observation that the genomic repeats that cause most of the complexity in a genome represent just a small fraction of the size of the genome. Only mate-pairs that span these, relatively rare, complexity hot-spots are useful during assembly. While Table 1 highlights the mate-pair statistics discussed here, Table 2 provides a comprehensive set of statistics for all read/mate-pair-length combinations studied.

**Table 2:** More complete mate-pair statistics

K-mer	MP Size(nt)	PathLenMatch*^a^*	CrossFork*^b^*	MatchSeq*^c^*	Unique*^d^*	Usable*^e^*	ComplReduc*^f^*
100	400	99.96	0.78	91.84	83.33	0.48	57.97
	2000	98.99	2.90	63.82	59.71	1.03	58.87
	6000	96.86	6.05	62.21	61.42	2.20	60.50
	8000	95.85	7.41	62.01	61.60	2.69	60.13
	35000	84.60	20.43	60.89	62.21	6.55	58.27

50	400	99.68	1.87	72.72	64.92	0.75	47.22
	2000	97.72	4.58	61.08	64.42	1.69	46.84
	6000	93.72	9.18	62.42	67.93	3.63	47.09
	8000	91.87	11.16	62.75	68.69	4.39	46.98
	35000	72.98	29.16	62.74	69.43	8.98	45.17

35	400	99.5	2.68	68.5	65.67	1.06	35.87
	2000	96.45	6.56	63.66	69.57	2.82	34.17
	6000	90.51	13.30	66.05	72.61	5.77	33.90
	8000	87.84	16.04	66.47	72.99	6.77	33.66
	35000	62.29	38.43	66.47	73.91	10.78	31.62

In the table below, a library of 50,000 mate-pairs of a particular length was applied to each of the graphs for each of the 391 genomes listed in Additional file 1. The values refer to average percentages. The first four columns refer the percentages retained by successive filtration steps used to identify and remove unusable mate-pairs: *^a^*% which had a shortest path of the prescribed length between end sequences, *^b^*% which crossed forks, *^c^*% which shortest path matched insert sequence, and *^d^*% which had a unique shortest path. The final two percentage values (*^e^*% of mate-pairs that were usable, and *^f ^*% reduction in finishing complexity) refer to overall percentages across all 50,000 mate-pairs for each category.

As a corollary to the above observation, the value of mate-pairs is maximized only once sufficient coverage is achieved to ensure that virtually all resolvable repeats are adequately spanned. The necessary number of mate-pairs is a function of the genome size *G *and the *k*-mer length *k *used for graph construction (roughly 10*G*/*k*; see Methods for details).

### 'Localized complexity' is common in short-read assembly graphs

**Figure 1:**
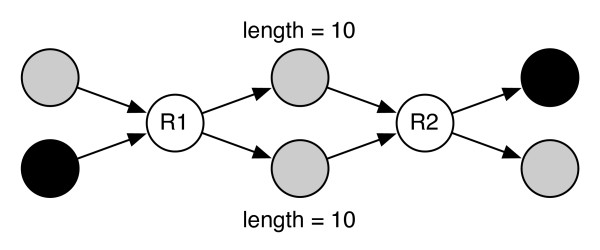
**An assembly 'bubble'**. An assembly 'bubble' that complicates repeat resolution with mate-pairs. Shaded nodes are non-decision nodes (in- and out-degree equal to 1). The nodes *R*1 and *R*2 are decision nodes (repeats). There are two possible paths of the same length from one end of the mate-pair to the other (black nodes), leading to ambiguity in the graph traversal.

A surprising result of our study has been the fact that, for the majority of the genomes studied, assembly ambiguity can only be decreased up to a certain point irrespective of depth of coverage and library size, i.e. mate-pair information appears to have limited value in resolving repeats. Closer inspection of the assembly graphs reveals a common motif that limits the applicability of mate-pair information. Specifically, we find pairs of repeat nodes (*R*_1_, *R*_2_), separated by two or more non-decision nodes of equal lengths. Frequently, chains of such patterns (commonly called 'bubbles') can be found in many genomes. Assembly bubbles are difficult to resolve with mate-pairs because multiple equal-length paths can be found between the endpoints of mate-pairs that span the bubble structure. Thus mate-pairs which span bubbles provide no information on the order in which the intermediate non-decision nodes (between *R*_1 _and *R*_2_) need to be visited. An example is shown in Figure 1. These regions of 'localized complexity' can only be resolved by mate-pairs that tightly span exactly one of the two repeats. As we will show later, this phenomenon is common in bacterial genomes and highlights the need to tune mate-pair libraries to the specific repeat structure of each genome.

In order to estimate the extent of genomic complexity introduced by the bubble pattern described above, we define a measure of the localized complexity of a genome as follows. For each repeat node, *v *∈ *G*, we define the node to be 'trivial' if all of its successor nodes have distinct lengths, and 'non-trivial' otherwise. The motivation for this nomenclature is that if *v *has multiple successors of the same length, it is likely that *v *fits the description of repeat *R*_1 _in the above stated example of an assembly bubble, and will be difficult to resolve without targeting at least one mate-pair to barely span its length. On the other hand, if all successors of *v *are of different lengths, *v *cannot fit the description of *R*_1 _in the above example, and the proper traversal order of *v *can likely be resolved using mate-pairs of arbitrary (longer) length. Thus we define the localized complexity of a genome, C-Statistic, to be the percentage of the finishing complexity of the genome contained in non-trivial nodes (C-Stat(*G*) = 100 , where *N *is the set of non-trivial nodes in the assembly graph, *S *is the set of all nodes in the assembly graph, and *C*_*v *_is the contribution of node *v *to the finishing complexity of the genome (see Methods for details on finishing complexity).

**Figure 2:**
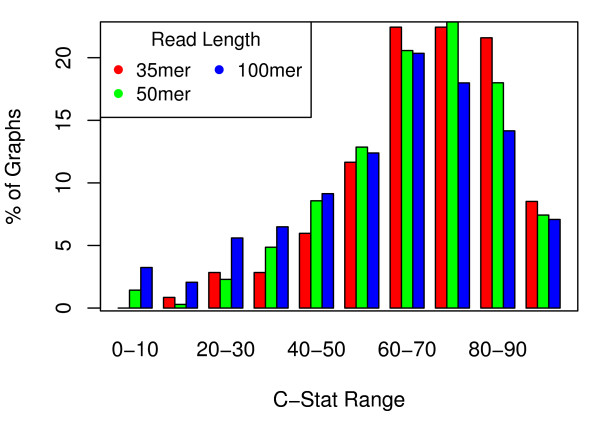
**C-statistic across 391 bacterial genomes**. The percentage of short-read (35, 50, and 100-mer) graphs with C-Statistic in a particular range. Here we see that in about 60% of the graphs created from short-reads, 60-90% of the finishing complexity is contained in repeats that are difficult to resolve using mate-pair information.

As seen in Figure 2, roughly 60% of the 35, 50, and 100-mer graphs have a C-statistic ranging between 60 and 90. In other words, in about 60% of the graphs created from short reads, 60-90% of their total finishing complexity is contained within repeats that are difficult to resolve using mate-pair information. The amount of localized complexity appears to be significantly lower for graphs created from longer reads: for 250-mer graphs, the average C-Statistic was 48.58%, and for the 500-mer graphs the average C-statistic was 33.61%. This implies that longer reads may be critical if the goal of assembly is to reconstruct entire genomes in an automated fashion.

The existence of bubbles in assembly graphs has been noted before, and several approaches have been suggested for resolving such regions (see, e.g., Chaisson *et al*. [16]). Our results imply that any such methods will be of limited use unless the mate-pair libraries are tuned to match the repeat structure of the genome being assembled.

### 'Ideal' mate-pairs are short

We further explored the hypothesis that 'tuning' mate-pair libraries to accommodate a genome's specific repeat structure could lead to higher quality assemblies. All repeats in a genome were grouped according to size into bins of width 4*k*, where *k *is the read length. For each bin we calculated the fraction of the total finishing complexity (see Methods) of the genome that is due to the repeats from that bin. We then selected the two bins with the highest finishing complexities, and constructed mate-pair libraries that just span repeats in these bins by using inserts that were 3*k *longer than the average repeat size for each bin. We restrict our analysis to just two libraries for any given genome to match the setting commonly encountered in practice.

Mate-pairs selected in this fashion end up being roughly proportional in size to the read length, *k*, averaging from 4.5*k *to 6*k*. This is significantly shorter than commonly constructed long-range libraries (6,000 bp or longer).

**Figure 3:**
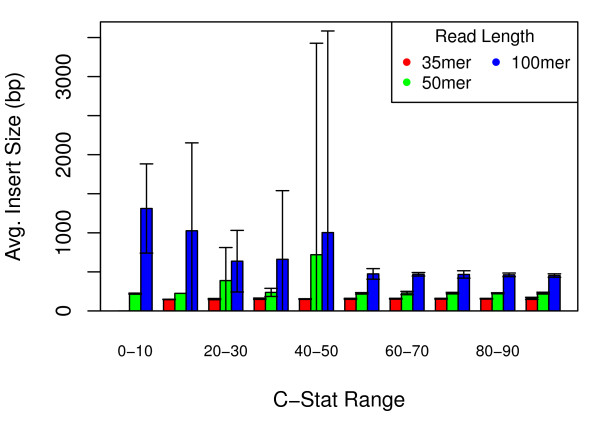
**'Ideal' mate-pair lengths across 391 bacterial genomes**. Average 'tuned' insert sizes for 35, 50, and 100-mer graphs, separated by C-Statistic. As can be seen, those graphs with a C-Statistic higher than 50 had a nearly uniform distribution and short insert sizes, while those with a lower C-Statistic had longer inserts and higher variance. Error bars represent standard deviation.

As expected, the tuned insert length is greater for graphs with less localized complexity (lower C-Statistic) than it is for graphs with greater amounts of localized complexity (higher C-Statistic). For 35, 50 and 100-mer graphs with a C-Statistic of at least 50, the mean ideal insert size was consistently short (4.5*k *to 6*k*), while genomes with a lower C-Statistic resulted in longer and more varied library sizes (see Figure 3). Increased variation in ideal mate-pair size for graphs with lower C-Statistic can likely be attributed to there being fewer graphs in this category (compare Figures 2 and 3).

These results are similar in spirit to, and complement, the EULER-PCR algorithm proposed by Mulyukov *et al*. [22]. In EULER-PCR, the assembler uses information about the repeat length (along with the known sequences on either side of the repeat) in order to minimize the number of primers and multiplex PCR experiments needed to resolve tangles in the graph. Essentially, the assembler is leveraging the repeat structure of the graph to find optimal sets of short mate-pairs to reduce manual finishing. In the context of producing mate-pair libraries via sequencing, we cannot target individual repeats, so we instead attempt to find the mate-pair sizes that are most likely to resolve a large fraction of a genome's repeats.

### 'Tuned' mate-pair libraries perform well in a simulated setting

To evaluate the effectiveness of the 'tuned' mate-pair libraries just described, we analyzed through simulations 360 bacterial genomes (each marked by an * in Additional file 1) across 5 different read lengths. (Several of the 391 genomes we originally analyzed required prohibitive computational resources and these genomes were excluded from the current analysis.) For each genome we compared the performance of the 'tuned' mate-pair libraries to a mixture of 2,000 and 8,000 bp libraries. The latter mixture deserves a brief explanation: the majority of genome projects (irrespective of sequencing technology, see e.g. [23,24] and jgi.doe.gov) rely on a mixture of library sizes, one of which is relatively long (possibly exceeding 10,000 bp and even as long as 40,000-50,000 bp in the case of fosmid libraries). As we cannot feasibly test all possible combinations of library sizes, we chose a combination that is reasonable and roughly matches the 'typical' scenario used in Sanger sequencing projects. In both cases (tuned libraries or long libraries) the depth of coverage was the same, and was chosen to ensure that all repeats can be adequately spanned (see Methods). For each genome we recorded the finishing complexity (see Methods) before and after the incorporation of mate-pair information, as well as the localized complexity (C-Statistic) of the genome.

**Figure 4:**
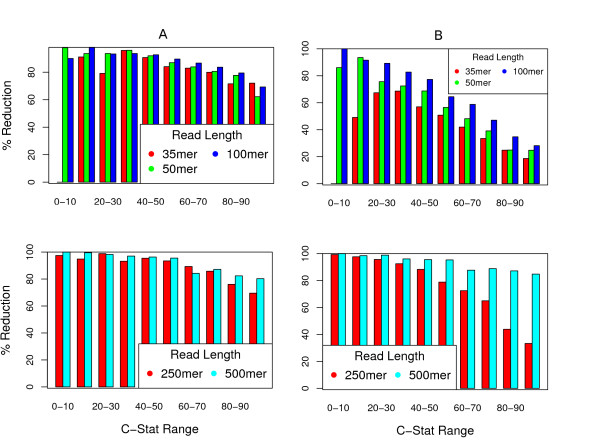
**Reduction in finishing complexity for 'tuned' vs. standard mate-pairs**. Graphs on left (*A*) depict the mean percent reduction in finishing complexity on graphs constructed from a particular k-mer size given a set of ideal ('tuned') mate-pair libraries (grouped according to the C-Statistic). The ideal libraries are graph-specific, but the smaller of the two libraries averaged between 4.5 *k *and 6 *k*, where *k *is the original *k*-mer size (read length) used to construct the graph. Graphs on right (*B*) depict the same statistics when using a mixture of two long libraries (2000 and 8000 bp).

The tuned libraries, which generally consisted of very short mate-pairs (see previous section of Results), vastly outperformed the long-range libraries on graphs constructed from short reads (35, 50, and 100 bp). The average reduction in finishing complexity was 82.66% when using tuned libraries, in contrast to only 47.44% when using a combination of long-range libraries (see Figure 4).

For a combination of two long mate-pair libraries, one can see a clear inverse relationship between reduction in finishing complexity and C-Statistic (two graphs on right in Figure 4). However, this relationship is greatly diminished for the tuned libraries (two graphs on left in Figure 4). This result is consistent with the observation that tuned library sizes are longer for the low complexity genomes, where chains of trivial repeats can often be resolved simultaneously by a single mate-pair.

While the results of simulations on 250-mer graphs were very similar to the results of simulations on the shorter-read graphs (tuned mate-pairs outperformed long mate-pairs), the performance improvement over our 'standard' library mixture decreases for 500-mer graphs (see bottom two graphs in Figure 4). This can be attributed to the fact that the standard insert size of 2000 bp is short (exactly 4*k*) with respect to the original read length for the 500-mer graphs, while it is long (exactly 8*k*) with respect to the original read length of the 250-mer graphs. This result indicates that the need for tuning mate-pair lengths to the genome structure may be unique to the use of very short reads.

### 'Tuned' mate-pair libraries improve performance when using off-the-shelf assembly software

In order to analyze the effectiveness of 'tuned' vs. long mate-pair libraries in a more realistic context, we created 30× coverage of 8 complete bacterial genomes (see Table 3) with 100 bp reads using MetaSim (see Methods). We then reassembled each genome given its set of reads and different combinations of long or tuned mate-pair libraries using SOAPdenovo v. 1.04 (see Methods). For each genome, one pair of tuned libraries was based on the 'ideal' lengths predicted by our 35-mer graphs (since we used SOAPdenovo's maximum *k*-mer length of 31 bp) and another pair was based on the ideal lengths predicted by our 100-mer graphs (since our reads were 100 bp long). See Table 3 for ideal mate-pair lengths for each genome.

**Table 3:** Genome-specific mate-pair lengths used in SOAPdenovo assemblies

Organism	K-mer Size	t1 (bp)	t2 (bp)
*Acinetobacter baumanii*	35	165	276
	100	454	1708

*Bacillus anthracis*	35	157	451
	100	464	839

*Bacteroides thetaiotamicron*	35	159	297
	100	462	1759

*Deinococcus radiodurans*	35	145	745
	100	485	930

*Mycobacterium tuberculosis*	35	155	288
	100	491	824

*Rickettsia prowazekii*	35	170	288
	100	467	N/A

*Staphylococcus aureus*	35	154	436
	100	461	943

*Vibrio parahaemolyticus*	35	161	298
	100	440	N/A

'Tuned' mate-pair lengths used when performing SOAPdenovo assemblies. The values t1 and t2 refer to the best and second best mate-pair lengths respectively. These values are based on determination of 'ideal' mate-pair sizes for the 35 and 100-mer graphs (as described in Results).

We found that in all but two of the cases, the pair of tuned libraries predicted by our 35-mer graphs were most effective at improving assemblies in terms of increasing average and maximum contig size, producing fewer total contigs, and increasing N50 contig size (see Table 4). These libraries consisted of one library of length at most 165 bp and a second of less than 800 bp in all cases. For the two cases in which the tuned libraries did not perform best, a single 200 bp library performed best. However, when splitting read-coverage between libraries of size 200 and 6000 bp, performance always suffered in comparison to the 200 bp library alone. These findings highlight our previously stated result that shorter mate-pairs are generally more helpful in assembly of prokaryotes than are longer mate-pairs. Interestingly, the tuned libraries predicted by our 100-mer graphs typically perform quite poorly despite the fact that the assembly is built from 100 bp reads. This indicates that despite the 'read-threading' procedure used by SOAPdenovo, information initially contained in a read is being lost when it is broken into shorter *k*-mers (31 bp is the maximum *k*-mer size for SOAPdenovo). There is little to no overlap between SOAPdenovo's assembly algorithms and the methods we have described for applying mate-pairs to ideal graphs in our simulations; yet tuned libraries perform better in both situations, indicating that they are capturing a fundamental property of the genome structure.

**Table 4:** Results of SOAPdenovo assemblies

Organism	Libs	Num	Total Size	Min Size	Max Size	Avg Size	Median Size	N50
*Acinetobacter baumanii*	200	6327	4064963	68	37142	642.48	100	7905
	200+6000	8192	4003114	33	29829	488.66	100	2455
	165	6991	4032232	40	18098	576.77	100	3653
	**165+276**	**6014**	**4069844**	**26**	**48396**	**676.73**	**100**	**12063**
	454	8544	4015574	33	20322	469.99	100	2919
	454+1708	8038	4069168	33	21003	506.24	100	3425

*Bacillus anthracis*	200	9102	5928608	33	48738	651.35	100	7943
	200+6000	10302	5884980	23	28576	571.25	100	4948
	157	10654	5865861	21	15749	550.58	100	2872
	**157+451**	**9184**	**5956270**	**12**	**43427**	**648.55**	**100**	**9257**
	464	12716	5831216	33	14895	458.57	100	2724
	464+839	16578	5686718	32	12862	343.03	100	1204

*Bacteroides thetaiotaomicron*	200	11092	7094608	34	49008	639.61	100	8114
	200+6000	12638	7044065	33	31728	557.37	100	4418
	159	12993	7033473	94	19688	541.33	100	2941
	**159+297**	**11024**	**7133929**	**38**	**63771**	**647.13**	**100**	**10826**
	462	15383	6975016	32	15352	453.42	100	2747
	462+1759	13948	7106347	32	22002	509.49	100	3703

*Deinococcus radiodurans*	**200**	**5222**	**3053941**	**11**	**57264**	**584.82**	**100**	**7449**
	200+6000	6436	2975712	33	10788	462.35	100	2142
	145	6166	2977456	21	12433	482.88	100	1952
	145+745	7033	2957901	16	13154	420.57	100	1715
	485	7822	2962094	22	11751	378.69	100	1744
	485+930	9452	2892284	26	11512	306	100	913

*Mycobacterium tuberculosis*	**200**	**8906**	**5075368**	**31**	**25729**	**569.88**	**100**	**6301**
	200+6000	11220	4978074	23	17275	443.68	100	2095
	155	10022	4996604	25	18585	498.56	100	2520
	155+288	12160	4931391	33	12018	405.54	100	1544
	491	12851	4959477	32	11915	385.92	100	1929
	491+824	12285	5065937	33	19438	412.37	100	2368

*Rickettsia prowazekii*	200	1933	1275800	58	34779	660.01	100	8633
	200+6000	2455	1254405	33	21498	510.96	100	2782
	170	2064	1258665	100	16628	609.82	100	4242
	**170+288**	**1846**	**1275767**	**38**	**47307**	**691.1**	**100**	**11463**
	467	2639	1255736	33	16084	475.84	100	2833

*Staphylococcus aureus*	200	5099	3240933	20	34464	635.6	100	7206
	200+6000	6428	3177307	33	18574	494.29	100	2465
	154	6066	3202427	16	17004	527.93	100	2602
	**154+436**	**5089**	**3263973**	**20**	**62134**	**641.38**	**100**	**9162**
	461	6940	3184253	33	20378	458.83	100	2783
	461+943	6466	3242406	33	19545	501.45	100	3498

*Vibrio parahaemolyticus*	200	3396	2151560	100	48275	633.56	100	8169
	200+6000	4238	2119692	34	16163	500.16	100	2697
	161	3897	2129657	65	16127	546.49	100	3125
	**161+298**	**3350**	**2159809**	**35**	**45188**	**644.72**	**100**	**10626**
	440	4304	2128652	32	17300	494.58	100	3567

Contig statistics for various assemblies using SOAPdenovo with 'tuned' and 'standard' mate-pair libraries. The standard sizes used were always 200 bp inserts or a combination of 200 bp and 6000 bp inserts. Tuned sizes varied according to the prediction of ideal mate-pair sizes provided by analysis of our 35-mer and 100-mer graphs (corresponding to sizes listed in Table 3). All assemblies used 30× coverage with 100 bp reads created by MetaSim. The best assembly (in terms of N50 contig size) for each organism is bold-faced.

## Discussion

There are a few difficulties we encounter when attempting to apply the exact quantitative analysis we describe above (and in Methods) to real assembly graphs. First, while our ideal graphs are unambiguously directed, real assembly graphs are inherently bi-directed since DNA is double-stranded. Therefore, it is not possible to directly transform graphs created from real data into unambiguously directed graphs so that the exact finishing complexity (by our definition) can be computed. Specifically, our quantitative analysis requires that we know the in-degree and out-degree of a particular node, yet the direction in which the edges are traversed in a real graph is often not known until the assembly process. Additionally, since edge multiplicities in real sequencing experiments must be estimated based on various metrics such as depth of coverage and errors occur during sequencing, real assembly graphs are often disconnected and certainly not fully Eulerian, further limiting the exact analysis described.

Nonetheless, the strategy for choosing the 'tuned' mate-pair sizes should be applicable to real genome projects. Note that our strategy for tuning the library sizes requires information about the amount of genomic complexity implied by a particular set of repeats of similar size, i.e. it is not sufficient to simply estimate the number and size of repeats from a genome assembly. Instead, the assembly graph (commonly output by many modern assemblers) needs to be analyzed in more detail. As we already mentioned, real assembly graphs have a different structure than the graphs used in our simulation. In order to accommodate the non-Eulerian nature of real assembly graphs, the methods we proposed can be converted to a Chinese Postman traversal setting, i.e., find a minimum-length tour of a non-Eulerian graph that covers each edge at least once. An efficient algorithm for solving this problem on bi-directed de Bruijn graphs has been recently published [25]. The resulting Chinese Postman tour implies an underlying Eulerian graph (which is implicitly constructed while solving the traversal problem) within which the algorithms described in our work can be applied. Specifically, within this implied graph we can compute, for each repeat, the corresponding finishing complexity which, together with the repeat size (already known from the original assembly graph), can be used as outlined in the Results (subsection titled "Ideal mate-pairs are short") to heuristically determine appropriate mate-pair sizes. In future work we plan to build a software package that performs this analysis for commonly used genome assemblers.

## Conclusions

Our results demonstrate that dramatic improvements in the quality of prokaryotic genome assemblies can be achieved by tuning mate-pair sizes to the actual repeat structure of a genome, suggesting the possible need to change the way that such sequencing projects are designed. In many sequencing projects, library sizes are chosen based on the ease with which certain size libraries can be effectively constructed in the lab. Virtually always, the mate-pair libraries are constructed at the beginning of the project, before having an opportunity to evaluate the repeat structure of the genome being sequenced. This 'one-step' approach was unavoidable in the case of Sanger sequencing where mate-pairs were a by-product of the sequencing process (sequencing would ultimately cost the same whether or not mate-pairs are generated).

In the case of next-generation sequencing technologies, however, the protocols were originally developed for creating unpaired reads, and the generation of mate-pairs generally increases the costs (in terms of prep time, reagents, and machine runtime) of sequencing. In this context, the two-tiered approach we propose - first generate an assembly of the genome with unpaired reads and evaluate the repeat structure of the genome; then generate the mate-pair libraries that provide most information towards the resolution of repeats in the genome being assembled - is not only possible, but likely more cost-effective in the long run. The tuned mate-pair libraries produced by this process will be more usable by the assembler, leading to better assemblies (longer correct contigs), which will dramatically reduce the cost of downstream manual finishing.

Across all of the genomes examined, as read lengths get shorter, the overall efficacy of mate-pairs in reducing finishing complexity is almost always diminished regardless of the amount of localized complexity present in the genome. This result is supported by the empirical observation that genome assemblies constructed from short-read data (e.g. ABI Solid or Illumina) are typically substantially more fragmented than those constructed with longer reads (454 or Sanger) [1], further underscoring the need to develop affordable sequencing technologies that generate long reads.

Many advances have been made in the field of automatic genome assembly in the past several years. Here we have shown that a novel, two-tiered approach to sequencing projects (along with the development of technologies which can reliably and affordably produce mate-pairs of a desired size) can greatly improve automation. However, even under ideal circumstances (idealized graphs, error-free reads, perfect coverage, and tuned mate-pair sizes), we still found ourselves unable to produce completely gapless assemblies in many cases. On average, approximately 17% of the original finishing complexity of a given genome still remained after applying mate-pairs. Thus, although the automation process continues to improve, the development of high-throughput and cost-effective approaches for genome finishing will also be of great importance in the years to come.

## Methods

### Idealized repeat graphs

A commonly used formulation of the assembly problem, based on de Bruijn graphs, was introduced by Pevzner *et al*. [4]. Specifically, a de Bruijn graph of order *k *is a graph that contains a node for every (*k *- 1)-length string (referred to as a (*k *- 1)-mer) over a given alphabet, and that contains an edge for every pair of (*k *- 1)-mers that overlap by exactly (*k *- 2) letters (the two nodes and the edge thus represent one of all possible *k*-mers). In the context of assembly, we focus on the sub-graph of the de Bruijn graph that corresponds to the *k*-mers that are actually present in the genome being assembled (for simplicity we will refer to this sub-graph as the de Bruijn graph as well).

We constructed de Bruijn graphs from the complete genome sequences of 391 prokaryotes (obtained from ftp://ftp.ncbi.nlm.nih.gov; see Additional file 1 for genome identifiers) as follows. We first chose a *k*-mer size, *k *(values 35, 50, 100, 250, 500 bp were used to be representative of read-lengths achievable through a range of modern sequencing technologies). For each (*k *- 1)-mer present in the genome, a node was created (without repetition of nodes for repeated (*k*-1)-mers). For each *k*-mer present in the genome, a directed edge was created connecting the node representing its first (*k *- 1) letters to the node representing its last (*k *- 1) letters. If a *k*-mer was repeated in the genome, then a number of edges equal to the number of instances of the *k*-mer were created. Thus we created 'ideal graphs' that are representative of perfect sequencing experiments (exactly one read for every *k*-length substring in the genome and no sequencing errors). In a real sequencing experiment, due to the double-stranded nature of DNA, each read and its reverse complement must be considered, resulting in a bi-directed graph. For the sake of simplicity, our graphs were constructed using only reads in the forward direction.

A series of lossless simplifications were applied to the resulting de Bruijn graphs as described by Kingsford *et al*. [2]. The first is a standard path compression in which if there exists a pair of nodes *u *and *v*, where *u *is the only predecessor of *v*, and *v *is the only successor of *u*, the nodes are combined into a single node with the same predecessors as *u*, and the same successors as *v*. The new node is labeled with the concatenated sequences of nodes *u *and *v *(accounting for the overlap between these sequences). A second type of compression is based on the idea that all areas of the de Bruijn graph whose cycle decomposition is a tree has a unique Eulerian tour, and can therefore be collapsed into a single node representing the sequence given by this traversal. The third technique iteratively 'unzips' half-decision nodes (those nodes with only one predecessor but multiple successors, or vice versa) by duplicating them and then performing standard path compression on the resulting unambiguous areas of the graph traversal.

**Figure 5:**
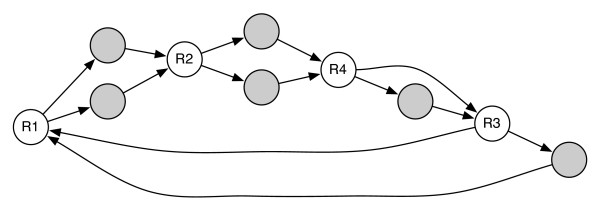
**A simplified de Bruijn graph**. A small de Bruijn assembly graph after the simplification process is complete. The nodes R1, R2, R3, and R4 are repeats (decision nodes), and the shaded nodes are non-decision nodes. Note that non-decision nodes can only reside sandwiched between repeats.

The simplified graphs are significantly smaller in terms of number of nodes and edges, yet the same set of Eulerian traversals exist before and after simplification since the transformations simply condense unambiguous paths into single nodes. Additionally, these graphs provide a very clear picture of the genome's 'repeat structure' (see Figure 5): Every node in the refined graph is either a repeat itself (has several pairs of in -and -out edges) or it is a non-repeat (has exactly one pair of in -and -out edges) that resides sandwiched between two repeats. Thus the length of each repeat, as well as the number of times it appears in the genome, can be clearly seen in the refined graph.

### Simulating mate-pairs

We simulated mate-pairs along the original genome sequence using the following procedure. Given a library size *l*, we chose a mate-pair specific size *d *at random from a Gaussian distribution with mean *l *and standard deviation 0.1*l*. A starting point s along the genome was selected uniformly at random, and two reads of length *k *(same as the value used in constructing the graph) were selected starting from positions *s *and *s *+ *d *- *k*. The reads were identified within the graph through a simple look-up, and the corresponding nodes were paired. In simulations involving mate-pairs of varying lengths, an equal number of mate-pairs of each length was used.

Throughout the analysis we assume to know the exact length *d *for each mate-pair, while in a practical setting only an approximate distance between mated-reads is known. Since there may be many paths of approximately the distance implied by a mate-pair, using our assumption can eliminate uncertainty as to whether a mate-pair implies a truly unambiguous path. Thus choosing not to model the uncertainty of mate-pair sizes allowed us to focus on understanding the effect of insert size on repeat resolution, rather than becoming stymied by ambiguity or resorting to heuristics aimed at choosing the 'most-likely' of potentially many paths implied by the mate-pair. In practice, this approach also allows us to use a great many mate-pairs in our simulation that might not have been usable by a real assembler. Although we do not model uncertainty about mate-pair sizes, we do take into account the variability in mate-pair sizes within one library that occurs during real mate-pair creation by choosing the mate-pair size from a Gaussian distribution (as described above).

### Repeat resolution through mate-pairs

We map each end of a mate-pair to a specific node in the graph. Because the graphs and mate-pairs are constructed from the same known sequence, the coordinates of each node in the assembly graph with respect to the reference genome is known, and this information is used to determine the placement of the mated reads within the graph. In a practical setting, this placement of mated reads can be determined by finding all good alignments (or some subset of the best of these alignments) of the mated sequences to nodes in the graph, then choosing a placement that most closely reflects the length of the insert based on some path-finding heuristic.

We check the graph for a shortest path that both connects the endpoints of a mate-pair and is consistent with the exact insert length. If a mate-pair cannot be mapped to a shortest path in the graph it is eliminated from further consideration. Mate-pairs whose ends map to a same node were excluded from further analysis as they can clearly provide no additional connectivity information. Similarly, we excluded from further analysis those mate-pairs whose ends mapped to two adjacent nodes in the graph. Since such mate-pairs do not span repeats, we see no clear way to use them for repeat resolution. Additionally, if more than one shortest path connects the ends of a mate-pair, there is ambiguity as to which path is the correct one, so the mate-pair is not used. Essentially, only mate-pairs connected by a unique shortest path that is exactly consistent with the known insert length are used for repeat resolution.

Mate-pairs fitting the above criteria are considered useful for disambiguating repeats. Specifically, these mate-pairs indicate that the Eulerian tour through the graph that is consistent with the correct reconstruction of the genome must contain the shortest path connecting their endpoints, thereby eliminating the uncertainty introduced by decision nodes (forks) along these paths.

More sophisticated heuristics for using mate-pair information could be applied here (several have been previously proposed as outlined in the Background section). We rely on a shortest path heuristic in order to ensure computational tractability since, in the general case, finding a path of a given length within a graph is *NP*-hard [15]. While this heuristic may fail in certain circumstances (i.e. a unique path, consistent with the mate-pair length, exists between the two ends yet it is longer than the shortest path), our results show that, in practice, the vast majority of mate-pairs have lengths consistent with a shortest path between the mated sequences (see Results). Thus more sophisticated heuristics should only have a limited impact. In addition, similar shortest path approaches have been previously used in genome assembly (see, e.g. Medvedev *et al*. [21]).

### Finishing complexity of the graph

In order to quantitatively characterize the benefit of mate-pair information for repeat resolution, we define a new measure of assembly complexity. Our measure, which we call 'finishing complexity', estimates the number of manual fishing experiments that would be required in order to correctly resolve all repeats and reconstruct a complete and correct version of a genome given the current state of the assembly graph. By using such a measure we assume that the primary goal of the assembly process is to automatically reconstruct as complete a picture of the genome sequence as possible. This measure may not directly apply to other contexts; e.g., in a project that only attempts to correctly reconstruct the genes, rather than the full sequence, of an organism.

A straightforward strategy for manually resolving a repeat is to choose an arbitrary in-edge of the repeat and try pairing it with each possible out-edge using targeted experiments (e.g. through targeted PCR) until the correct pairing is found, at which point both the in-degree and out-degree of the repeat are effectively reduced by 1. This process is then repeated for the remaining edges. The last pairing is implicit, as only one in-edge and one out-edge remain. Thus we define a node's finishing complexity to be , where *a *can be either the in-degree or out-degree of the node. Since our graphs are Eulerian, the in-degree and out-degree of any particular node are always equal. The total finishing complexity of the graph is simply the sum of the finishing complexities of all nodes.

### Determining the optimal number of mate-pairs

Our results on 'localized complexity' (see Results) indicate that it is frequently necessary to target mate-pairs to tightly span repeats of a particular size if we wish to produce mate-pairs that can imply truly unambiguous paths. However, simply picking an insert size that can potentially span repeats of this size is not sufficient to ensure that (when mate-pairs are randomly generated) all such repeats will be disambiguated by the resulting library. It is also necessary to generate sufficient coverage such that each instance of every repeat of the targeted size is actually spanned by at least one mate-pair. This is especially important if very short mate-pairs are being used.

By applying Poisson statistics, we can see that a level of coverage of approximately 10 at the read level is sufficient to virtually ensure that at least one mate-pair will span every instance of each repeat in the genome. Here we assume that coverage of the genome is uniform, which is a reasonable assumption, although it may not hold true for all current data sets. Specifically, let *X *be a random variable denoting the number of mate-pair arrivals across a sequence of any given *k *nucleotides in the genome. Let *λ *be the mean arrival rate for mate-pairs (coverage), which can be achieved by creating *λ*(*G*/*k*) mate-pairs, where *G *is the length of the genome in nucleotides and *k *is the original *k*-mer length used for construction of the graph. If we create 10(*G*/*k*) mate-pairs in total, then *λ *= 10, and the following holds:

Thus if we create 10(*G*/*k*) mate-pairs, we virtually guarantee at least one mate-pair arrival across any stretch of *k *nucleotides in the genome. Since no nodes in the graph are shorter than *k*, there should be at least one mate-pair starting in each node that directly leads into a repeat (with at least one additional mate-pair for each additional traversal of the node), and the determination of 'ideal' insert size (see Results) should guarantee that the other end of the mate-pair is just on the other side of the repeat (for the repeats of the size being targeted).

### SOAPdenovo assemblies

For each of 8 bacterial genomes to be assembled (see Table 3), 30× coverage in 100 bp reads were created using MetaSim v. 0.9.1 with an empirical error model derived from the 80 bp model provided by MetaSim (assuming the last 21 bp have the same error characteristics). Several sets of mate-pairs were constructed for the various assemblies, chosen to be reflective of either standard libraries, 'ideal' libraries (see Results) for a 35-mer graph, or ideal libraries for a 100-mer graph. The mate-pair lengths were distributed normally around the mean with a standard deviation of 10%. SOAPdenovo v. 1.04 was run with option -K31 (the largest *k*-mer size allowed by SOAPdenovo) and configuration options reverse_seq = 0 (standard mate-pair orientation), asm_flags = 3 (try harder to build large contigs), pair_num_cutoff = 2 (minimum number of mate-pairs required to link contigs), map len = 60 (minimum length match between a read and a contig during scaffolding).

## Authors' contributions

JW, CK, and MP all contributed ideas and participated in writing this article. MP conceived the study. JW and MP primarily designed and performed the experiments. CK contributed idealized graphs. All authors read and approved the final manuscript.

## Supplementary Material

Additional file 1 **A list of the 391 genomes used in the study, along with their identifiers**. The 360 genome subset of these that are discussed in the Results subsection regarding simulated comparison of 'tuned' vs standard mate-pair libraries are each marked with an asterisk (*). Click here for file
